# The development and psychometric properties of the Arabic version of the child oral health impact profile-short form (COHIP- SF 19)

**DOI:** 10.1186/s12955-017-0796-4

**Published:** 2017-11-13

**Authors:** A. A. Arheiam, S. R. Baker, L. Ballo, I. Elareibi, S. Fakron, R. V. Harris

**Affiliations:** 1 Department of Health Services Research, University of Liverpool, Liverpool, UK; 2 Unit of Dental Public Health, School of Dentistry, University of Sheffield, Sheffield, UK; 3 Department of Community and Preventive Dentistry, Faculty of Dentistry, University of Benghazi, Benghazi, Libya

**Keywords:** Quality of life, Oral health, Reliability, Validity, Arabic, Children

## Abstract

**Background:**

This study aims to cross-culturally adapt the original English-language COHIP-SF 19 to Arabic culture and to test its psychometric properties in a community sample.

**Methods:**

The Arabic COHIP-SF 19 was developed and its psychometric properties were examined in a population-based sample of 876 schoolchildren who were aged 12 years of age, in Benghazi, Libya. The Arabic COHIP-SF 19 was tested for its internal consistency, reproducibility, construct validity, factorial validity and floor as well as ceiling effects. A Mann-Whitney U test was used to compare the mean scores of COHIP-SF 19 by participants’ caries status and self-reported oral health rating, satisfaction and treatment need.

**Results:**

The Arabic COHIP-SF 19 was successfully and smoothly developed. It showed an acceptable level of equivalence to the original version. Overall, the internal consistency and reproducibility were acceptable to excellent, with a Cronbach’s alpha of 0.84 and an intra-class correlation coefficient (ICC) of 0.76. All hypotheses predefined to test construct validity were confirmed. That is, children who had active dental caries, and who rated their oral health as poor, were not satisfied with their oral health or indicated the need of treatment had lower COHIP-SF 19 scores (*P* < 0.05). Floor or ceiling effects were not observed. The exploratory Factorial analysis suggested a 4-component solution and deletion of one item.

**Conclusion:**

The Arabic COHIP-SF 19 was successfully developed. The measure demonstrated satisfactory reliability and validity to estimate OHRQoL in a representative sample of 12-year-old schoolchildren.

## Background

Oral health-related quality of life (OHRQoL) is a multidimensional, patient-centered subjective measure of functional and psycho-social impacts of oral health [1]. Recently, a new definition of oral health has been adopted by the Dental Federation General Assembly has acknowledged psychosocial function as a core element of oral health which a multifaceted construct [2]. This movement comes as no surprise since the psychosocial impacts of oral health have been the center of attention in the dental literature for some time now, in recognition of a paradigm shift in defining oral health needs and outcomes from a narrow biomedical to a wider biopsychosocial approach [3]. Many OHRQoL measures have been developed and used for oral health assessment, to supplement conventional clinical indicators [3, 4]. Amongst the various important theoretical, political and practical applications of OHRQoL measures [5], their use in epidemiological surveys has become increasingly popular [3, 6, 7]. The useof OHRQoL measures has huge implications for oral health services planning, evaluation and allocation of resources and decision making [8–11]; leading in due course to more efficient service planning [3, 4].

Dental caries is a major public health problem in many developing and developed countries, with significant impacts on quality of life, particularly among children [12, 13]. Dental caries can cause severe tooth pain [14, 15], sepsis and tooth extraction [16], and consequently significant impact on school attendance [17], and self-esteem of children [18]. Although many measures have been developed to assess OHRQoL among school age children [19, 20], the Child Oral Health Impact Profile (COHIP) stands out for being both suitable for children between 8 and 15 years of age, while also evaluating both positive and negative attributes of quality of life [21]. What is more, recently, a shorter version of COHIP (COHIP-SF19) has been developed using a confirmatory factor analysis [22]. Such short forms are appropriate for large surveys since they are less time consuming, easy to use and interpret and consequently more cost-effective [23].

However, since the initial development by Broder et al. in 2012, there has been very little published research on the cross-cultural adaptation and validation of COHIP-SF 19. To the authors’ knowledge, only one study has addressed this issue which was conducted in China [24]. Every time an OHRQoL measure is used in a different context or cultural group, it needs to be cross-culturally adapted and tested for its psychometric properties [25–27]. This procedure aims to ensure the suitability of the OHRQoL measure to the new context as well as its equivalence to the original measure. Herdman et al. (1998) [26] proposed a framework of six aspects of equivalence, defined in Table 1 (semantic, conceptual, item, operational, measurement and functional), to be considered when cross-culturally adapting quality of life questionnaires.

**Table 1:** Definition of aspects of equivalence according to Hardman (1998) [26], extracted from Saub et al. (2007) [35]

Equivalence	Definition
Conceptual	Ways in which different populations conceptualize health and quality of life (QoL) and the values they place on different domains of health and QoL.
Item	Concerns the way in which domains are sampled. Item equivalence exists when items estimate the same parameters on the latent trait being measured and when they are equally relevant and acceptable in both cultures.
Semantic	Concerned with the transfer of meaning across languages.
Operational	Refers to the possibility of using a similar questionnaire format, instructions, mode of administration, and measurement method (response format).
Measurement	Ensuring that different language versions of the same instrument achieve acceptable levels in terms of their psychometric properties – reliability, responsiveness, and validity.
Functional	The extent to which an instrument does what it is supposed to do equally well in two or more cultures.

Given that there are few child OHRQoL measures translated to Arabic (CPQ11–14 &C-OIPD) [19], and that no previous attempts have been made to develop an Arabic version of COHIP-SF 19; this study was conducted to cross-culturally adapt the original English-language COHIP-SF 19 to Arabic culture and to test its psychometric properties in population-based sample of 12-year-old schoolchildren in Libya.

## Methods and results

Ethical clearance and permissions for the study were obtained from ethics committee at the University of Liverpool and faculty of Dentistry at the University of Benghazi prior to data collection. Written informed consents were obtained from the parents/guardians. In this paper, the methods and results section are combined in one section to reflect the sequence of procedures employed in the cross-cultural adaptation and psychometric testing of Arabic COHIP-SF19, according to the guidelines proposed by Beaton et al. (2000) [25].

### Stage 1: Translation of the original COHIP-SF19

The original English-Language COHIP-SF19 (OV) was translated to the Arabic language using a rigorous forward-backward translation process. The OV was first translated into the Arabic language by two bilingual native Arabic speakers (an English language teacher and a dentist who lived for many in years in the UK). The translators worked independently and were pre-informed about the aim of the questionnaire and its target group. They were also requested to identify any ‘difficult to translate’ words. The two Arabic translations (T1 &T2) were then discussed with the research team to be consolidated in one Arabic version (T12). This process was then repeated the other way around. The Arabic version (T12) was translated into the English language by two native English speakers who speak Arabic fluently. Two independent translations (BT1 & BT2) were created, which were then discussed with the investigators to generate one English version (BT12). A committee of experts reviewed the translations and assessed its semantic equivalence to the OV [26], to approve a pre-final version of the Arabic COHIP-SF19. The committee of experts included a languages expert, the translators, two dentists and a dental researcher in the area of quality of life and two native English speakers [25].

None of the questionnaire items were found challenging for the translators or required a modification. The committee was satisfied with the Arabic version produced and no major or meaning related modifications were suggested. The pre-final version of Arabic COHIP-SF 19 was approved by the committee of experts. It comprised 19 items distributed over 3 conceptual subscales as following: Oral health (5 items), Functional well-being (4 items), and Socio-emotional well-being (10 items). A five-point Likert scale (‘never’ = 0, ‘almost never’ = 1, ‘sometimes’ = 2, ‘fairly often’ = 3, and ‘almost all of the time’ = 4.) was used to collect responses for all items. The question: ‘How often have you experienced oral impacts during the past 3 months?’ was posed at the outset of the questionnaire. After reversing the scoring of the 17 negatively-worded items, the total score ranged from 0 to 76, with the higher score indicating better quality of life.

### Stage 2: Testing of pre-final Arabic COHIP-SF 19

The pre-final Arabic COHIP-SF19 was tested for its conceptual, item and operational equivalence (Table 1). The questionnaire was piloted at the department of paediatric dentistry at the Faculty of Dentistry at the University of Benghazi. A separate group of 35 children who were not participants in the stage 3 study were asked to complete the questionnaire. Also, one-to-one interviews were conducted, in the presence of their parents, to explore children’s views regarding each item in terms of meaning, clarity of wording, relevance to oral health and its conceptual subscale and the response options. Based on the feedback received from the participants, a final Arabic COHIP-SF 19 was produced.

All the items were considered relevant and clearly understood. The domains were identical to the OV. No changes in the response options or the questionnaire format or mode of administration were suggested. The final Arabic COHIP-SF19 was pre-tested and produced.

### Stage 3: Psychometric properties of Arabic COHIP-SF 19

After the cross-cultural adaptation, it is highly recommended that the new version is tested for its measurement properties among its target population [25]. To do so, a cross-sectional study design was used to examine the psychometric properties of the Arabic COHIP-SF 19 in a population-based sample of 12-year-old Libyan schoolchildren. This study was part of a survey investigating oral health status and treatment needs in conflict-affected Libya and to compare these with pre-conflict data. Therefore, the survey aimed to collect data from a comparable sample size which was identified to be at least 800. Only procedures related to testing the psychometric properties of Arabic COHIP-SF 19 are reported here.

#### Study sample

The participants were 12-year-old school children registered in the sixth grade for the academic year 2016/17 in Benghazi, Libya. The sampling frame was a total of 12,761 children, with almost equal male and female distribution, registered in 40 state-run schools distributed over 8 main districts. The participants were recruited by using a multi-stage clustering random sampling technique, using the schools as the clustering unit. At the first stage, a proportional sample of schools was randomly selected from each district. At the second stage, children were randomly selected from each school. The random selection of schools and participants was chosen by using computer system. A minimum sample size of 400 had previously been identified to be sufficient for studies assessing reliability and validity [28]. In the present study, a total of 950 participants were recruited to take part, from 16 schools.

#### Questionnaire administration

The children’s schools were first approached to arrange for data collection. Informed consent was first sought from the parents which was sent to them through the school administration. Only participants with parental consent were included in the study. The Arabic COHIP-SF 19 was administered on a separate day by trained research assistants in quiet rooms in their schools, after explaining the aim of the study. Verbal assent was obtained from the children and implied by them returning completed questionnaires and attending the dental examination. The Arabic COHIP-SF 19 was provided along with another questionnaire covering oral health behaviors and sociodemographic information. Trained research assistants were available on demand at the research sites to aid the participants in completing the questionnaire. All participants took a maximum of 10 min to complete the questionnaire. The Arabic COHIP-SF 19 was administered again after 3 weeks to a sub-sample of 100 participants, randomly selected from 4 schools. This step was undertaken to allow the assessment of the measure’s reproducibility.

#### Clinical examination

Three dentists were trained and calibrated to carry out the clinical dental examinations. The training sessions were provided at the department of Community and Preventive Dentistry, University of Benghazi. Intra-examiner reliability and inter-examiner reliability were tested in a separate group of 12-year-old school children before commencing the data collection of the main study. Kappa coefficient ranged from 0.82 to 0.96. After completing the questionnaires, dental examination was conducted for all participants in a separate room under daylight while the participant was seated on an ordinary chair. The children were assessed for their oral health status and treatment needs according to WHO diagnostic criteria and forms, using disposable diagnostic kits. Dental caries experience was assessed at dentine level (Cavitation) using the DMFT and DMFS indices [29]***.***

#### Data analysis

Of 950 children recruited for the study, 876 participants provided complete questionnaires usable for analysis. All data analyses were conducted using SPSS software (IBM, Version 24). Internal consistency was assessed by calculating Cronbach’s alpha coefficient for the overall scale and for each subscale (Oral health, Functional well-being and Socio-emotional well-being). Cronbach’s alpha values ≥0.6 was considered as an acceptable level [30]. The intra-class correlation coefficients (ICC) were used to assess test**-**retest reliability. These were calculated for scores from the repeated administrations of the questionnaire. An ICC of 0.7 indicates an acceptable level of reproducibility [19].

Construct validity of Arabic COHIP-SF 19 was evaluated by examining measures of the discriminant and convergent validity [22]. These were examined against 4 predefined hypotheses [31], as following: lower COHIP-SF 19 scores would be observed among those who 1) perceived their oral health as poor; 2) were not satisfied with their oral health; 3) indicated the need for dental treatment; 4) had active dental caries (had more than one decayed tooth vs caries-free). To test these hypotheses, the participants were asked to answer 3 general questions on whether they were satisfied with their oral health (Satisfied VS not-satisfied), whether they perceived any need for oral health treatment (Yes VS No) and how they rated their own oral health (good/excellent VS poor). All hypotheses were tested by employing Mann-Whitney U test at *p* < 0.05.

An exploratory factor analysis (EFA) was conducted to test the factorial validity of items in the subscales defined in the original COHIP-SF19, using the varimax rotation and a strict cut-off of factor loading of >0.50 [32]. Item-impact values for the scale items were computed as the product of the mean score and percentage of participants generally had that impact (‘sometimes’ = 2, ‘fairly often’ = 3, and ‘almost all of the time’ = 4 responses on the item) [33]. The purpose of the item impact phase was to measure the prevalence and importance of the scale items in the Arabic culture.

The questionnaire was also tested for the existence of ceiling or floor effects by calculating the frequencies of participants who achieved the lowest or highest possible score. If more than 15% of participants achieved the lowest or highest possible score, the Arabic COHIP-SF 19 was considered to have floor or ceiling effects respectively [31].

#### Results of stage 3

##### Distribution and comparison by gender of Arabic COHIP-SF 19 scores

**Fig. 1:**
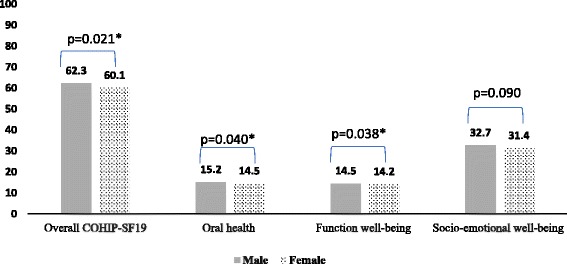
Comparison of overall COHIP-SF19 and its subscales by participants’ gender. Man-Whitney U test was used to compare the subgroups, * *P* ≤0.05

Table 2 shows the distribution of Arabic COHIP-SF19 scores and the subscales. The mean overall score was 61.13 (12.97) and ranged between 4 and 76. Scores for the overall scale and Oral health and Functional wellbeing subscales were significantly (*P* < 0.05) lower among female participants than that in males. The score of Social-emotional wellbeing subscale was also higher in males, although this was not statistically significant (Fig. 1).

**Table 2:** Summary reliability measures and descriptive statistics for Arabic-COHIP-SF 19 and subscale scores (*n* = 876)

Descriptive statistics	Overall scale, subscales (No. of Items)			
	Overall scale	Oral health well-being	Functional well-being	Socio-emotional well-being
Mean (SD)^b^	61.13 (12.97)	14.82 (4.58)	14.31 (3.01)	32.01 (8.45)
Range	4–76	0–20	0–16	0–40
Proportion of lowest possible score^a^	0 (0)	10 (1.1)	8 (0.9)	6 (0.7)
Proportion of highest possible score^a^	59(6.7)	185 (21.1)	548 (62.6)	244 (27.9)
Cronbach’s alpha	0.85	0.64	0.69	0.83
Alpha if an item is deleted	0.83–0.86	0.57–0.61	0.59–0.67	0.80–0.85

^a^Count (%), ^b^higher score indicates better quality of life

##### Internal consistency and test-retest reliability

The overall Cronbach’s alpha of Arabic COHIP-SF 19 was 0.85. For the subscales, Cronbach’s alpha was 0.65, 0.69 and 0.84 for the Oral health, Functional well-being and Socio-emotional well-being scales respectively. Generally, Cronbach’s alpha did not improve when any of the items were removed from the scale. The corrected item-total correlations were positive, ranging from 0.19 to 0.72. ICC for the overall scale and the subscales ranged between 0.70 and 0.76 (Table 2).

##### Construct validity

Table 3 presents comparisons of mean scores of Arabic-COHIP-SF 19 and its subscales by participants, caries status and oral health satisfaction, rating and perceived treatment need. The mean score of the overall scale and the subscales of Arabic COHIP-SF 19 were significantly higher among those who rated their oral health as ‘good/excellent’ than among those who perceived oral health as ‘poor/very poor’ (*p* > 0.001). The mean scores of Arabic COHIP-SF 19 and its subscales were significantly lower (*p* > 0.001) among children who did not feel satisfied with their oral health and who indicated the need of dental treatment (Table 3). Comparisons of mean scores across caries activity subgroups showed higher scores among caries-free children, which was statistically significant (*p* > 0.05) for the overall scale as well as Oral health and Functional well-being subscales but not for the Socio-emotional well-being subscale (Table 4).

**Table 3:** Comparisons of Arabic-COHIP-SF 19 and subscale scores by participants, caries status and oral health satisfaction, rating and perceived treatment need (*n* = 876)

	A-COHIP-SF 19 Overall scale, subscales			
	Overall (19)	Oral health well-being	Functional well-being	Socio-emotional well-being
Clinically assessed dental caries	Mean (SD)			
Caries free(*n* = 406)	62.13 (12.42)	15.37 (4.30)	14.51 (3.03)	32.25 (8.19)
Caries active (*n* = 355)	60.28 (13.38)	14.36 (4.76)	14.13 (3.04)	31.78 (8.67)
*P* value	0.033*	0.002**	0.031*	0.569
Participants’ satisfaction with oral health	Mean (SD)			
Satisfied (*n* = 665)	62.91 (11.56)	15.09 (4.43)	14.63 (2.67)	33.17 (7.57)
Not satisfied (*n* = 211)	55.56 (15.41)	13.98 (4.94)	13.27 (3.85)	28.31(9.94)
*P* value	≤0.001***	0.005**	≤0.001***	≤0.001***
Participants’ perceived oral health treatment needs				
Yes (393)	56.81 (14.54)	13.41(4.80)	13.58 (3.61)	29.81 (9.66)
No (483)	64.65 (10.29)	15.81 (4.05)	14.89 (2.35)	33.78 (6.83)
*P* value	≤0.001***	≤0.001***	≤0.001***	≤0.001***
Self-rated oral health				
Excellent/good (*n* = 584)	64.51 (9.84)	15.61 (4.03)	14.78 (2.43)	34.12 (6.54)
Poor (*n* = 292)	54.39 (15.60)	13.35 (5.18)	13.35 (3.73)	27.76 (10.10)
*P* value	≤0.001***	≤0.001***	≤0.001***	≤0.001***

Man-Whitney U test was used to compare the subgroups, * *P* ≤ 0.05, ** *P* ≤ 0.01, *** *P* ≤ 0.001

**Table 4:** Mean scores, item impacts and item-total correlations and EFA findings. Responses to how often have you experienced the following in last 3 months? (*n* = 876)

Item	Mean	SD	% of participants reported the impact	Item impact	Item total correlation	EFA component			
Oral Health—Well-being						1	2	3	4
Had pain in your teeth/toothache.	1.3	1.4	49.9	64.9	0.44		0.51		
discoloured teeth	0.8	1.3	24.5	19.6	0.36			0.62	
crooked teeth	1.1	1.6	33.1	36.4	0.39			0.59	
Had bad breath.	0.8	1.2	28.1	22.5	0.43			0.58	
Bleeding gums.	1.22	1.5	43.0	52.6	0.33				
Functional Well-being									
Had difficulty eating foods you would like to eat	0.7	1.3	23.5	16.5	0.48		0.69		
trouble sleeping	0.3	0.9	12.6	3.4	0.49		0.67		
difficultly saying certain words	0.3	0.9	8.6	2.6	0.36		0.61		
difficulty keeping your teeth clean	0.4	1.1	13.8	5.52	0.41		0.61		
Socio-emotional Well-being									
unhappy or sad	0.7	1.4	22.4	15.7	0.65	0.74			
Felt worried or anxious	0.7	1.6	21.1	14.8	0.72	0.78			
Avoided smiling or laughing	0.6	1.3	18.0	10.8	0.69	0.80			
Felt that you look different	0.5	1.2	15.2	7.6	0.69	0.84			
worried about what other people think	0.5	1.2	15.1	7.6	0.64	0.82			
Been teased or bullied by peers	0.5	1.2	14.8	7.4	0.64	0.79			
Missed School for any reason	0.2	0.7	6.8	1.4	0.34	0.61			
Not wanted to speak/read out loud in class	0.3	1.0	9.9	3.0	0.51	0.53			
Been confident	2.0	1.9	54.1	108.2	0.19				0.92
Felt attractive	2.0	1.8	56.2	112.4	0.20				0.91

##### Ceiling & floor effects

None of the participants achieved the lowest possible score (0) for the overall scale, whereas 6.7% of the participants achieved the highest possible score. For the subscales, the highest possible score was most commonly achieved in the Functional well-being subscale (67.5%). On the other hand, the numbers of those who achieved the lowest possible score were generally low in all subscales, and ranged between 6 and 10 participants (Table 2).

Table 4 presents the EFA and item-impact analysis. The EFA returned a 4-factor solution which explained 57% of data diversity. The item “bleeding gum” was eliminated. The item “pain” was grouped with Functional items. The change from the original 3-factor COHIP-19 was the addition of a new sub-scale which comprised of the items related to “Been confident” and “Felt that you were attractive”. Interestingly, as well as forming a separate sub-scale, these two items also showed the highest factor loadings, highest item impact scores and least total item correlation values (0.19 and 0.20, respectively). High item impacts were also observed for the dental pain and gingival bleeding Items.

## Discussion

The purpose of this study was to cross-culturally adapt the original English-language COHIP-SF19 to an Arabic cultural context and to test the psychometric properties of Arabic COHIP-SF19 in a population-based sample of Libyan schoolchildren. In reviewing the literature, only one study, conducted in China, has touched on testing COHIP-SF19 performance in a different culture [24]. To the authors’ knowledge, the present study is the first in an Arabic speaking country. The Arabic COHIP-SF19 was successfully developed and cross-culturally adapted, showing satisfactory equivalence and psychometric properties in comparison to the original English version.

The Arabic COHIP-SF19 demonstrated excellent ‘semantic equivalence’ to the original English version. Although it is not uncommon to face translation difficulties when cross-culturally adapt OHRQoL questionnaires from English to the Arabic language [34], the translation process in the current study was trouble-free. This observation can be traced back to the development of original COHIP-SF19 where items with content overlap were identified and eliminated [22]. The review committee was satisfied with the wording and the vocabulary used in the Arabic COHIP-SF19, which indicates excellent content and face validity.

The Arabic COHIP-SF19 showed satisfactory ‘item’, ‘conceptual’ and ‘operational’ equivalence. The participants in the pre-testing pilot reported that the questionnaire was clear, easy to use and relevant to its purpose. There was no need to modify the questionnaire’s instructions, mode of administration or response options. It is worth noting, however, that the study participants were all similar in education level and taught in Arabic language up to the Sixth-grade level. It is therefore possible that these findings may not apply to someone with limited literacy skills who may require assisted or interview mode of administration rather than self-completion [35].

The Arabic COHIP-SF 19 exhibited acceptable level of internal consistency as measured by Cronbach’s (0.85) which is comparable to that reported for the original English COHIP-SF19^22^ and for the Chinese version [24]. At the subscales level, only the Socio-emotional wellbeing scale showed acceptable value of Cronbach’s alpha (0.83). The Cronbach’s alpha values for Oral health and Functional well-being subscales were quite lower, although they were higher than those observed in the Chinese study [24]. However, items interrelatedness in these two subscales was acceptable (above the recommended level of 0.2 [36]). Therefore, low Cronbach’s alpha values, observed in the current study, may have something to do with the small numbers of items in Oral health and Functional well-being subscales [37]. The test–retest reliability for the overall scale of Arabic COHIP-SF19 was substantial, above the recommended threshold [38], indicating very good reproducibility for the Arabic COHIP-SF19 [19]. The ICC score for the overall scale was 0.76 which is and comparable to that found in the Chinese study [24] .

Construct validity was examined by testing the associations between Arabic COHIP-SF19 and clinical caries data and global ratings of oral health. Almost all predefined hypotheses were confirmed. The Arabic COHIP-SF19 was able to distinguish between subgroups according to their caries status. Our data show that caries active participants appeared to have lower COHIP-SF19 scores than their caries-free peers. In the current study, lower scores of COHIP-SF 19 were observed among those who rated oral health as ‘poor/very poor’, felt unsatisfied with their oral health and who perceived the need of dental treatment. These findings are in keeping with previous studies of COHIP-SF19 [22, 24], and suggest satisfactory construct validity.

EFA indicated that the Arabic version is characterized by 4 dimensions instead of the 3 dimensions suggested in the development study of original COHIP-SF19. The new dimension comprised 2 items related to self-image, which also showed high loading and item impact than other items in the scale. Current data does not allow for a plausible explanation to this finding but it may have something to do with variations in the characteristics of the study population [39]. The current study sample was recruited from community setting wherein oral health and function issues may not be as high as if the sample was recruited from a clinical setting. Unfortunately, previous studies of COHIP-SF19 did not report on item impact and factorial validity which precluded the comparison with our findings. Further research, however, is required to compare the 4- and 3-factor CHOHP-SF in community and clinical based samples.

The average score for Arabic COHIP-SF19 was relatively high (61.13 ± 12.97). This score is higher than that observed in the original study and among the Chinese children [22, 24], and suggests low oral health impacts among Libyan school children, which is not uncommon for children from Arabic speaking countries [40, 41]. In the current study, females were more likely to experience oral health impacts than their male counterparts. A similar trend has been observed in the dental literature on OHRQoL among children [34, 41–43]. Although it is well recognized in the general literature that females are more sensitive than males because of several biological, cultural, psychological, and social factors [44], gender differences in perception of OHRQoL should be taken in account when developing oral health interventions and programs.

The overall scale of Arabic COHIP-SF19 demonstrated a lack of floor and ceiling effects which reflects the validity and reliability of the response scale [31]. Interestingly, the ceiling effect existed in the subscales, which was frequently achieved in the Function well-being subscale. It is difficult to explain this observation, but it may have something to do with how the participants define what constitutes an optimum oral health. It is well recognized that individual’s appraisal of the quality of life is influenced by the extent to which expectations and goals are matched by experience [45]. The current study was conducted in a conflict-affected country which colors all aspects of live and hence the perception of oral health importance and impacts. Therefore, it could be the case that the participants gave higher ratings to functional impacts than they give to social and emotional impacts of oral health [40]. However, more qualitative work is required to further explore this phenomenon.

As for all cross-sectional studies, this study has some inherent limitations, specifically related to the evaluative performance of the Arabic COHIP-SF19. For example, it was impossible to assess the responsiveness of the Arabic COHIP-SF19, which has important implications for studies using OHRQoL as an evaluative outcome measure such as interventional studies and longitudinal observational studies aiming to improve oral health care [3]. In addition, the participants were limited to the 12-year-old age group, and hence age-related variations were not explored. Therefore, using longitudinal research design and including various age groups should be considered in future research.

## Conclusion

Using a comprehensive cross-cultural adaptation process, the original English language COHIP-SF 19 was successfully translated and adapted to the Arabic context. The Arabic COHIP-SF 19 is satisfactorily equivalent to the original version and is valid and reliable to estimate OHRQoL in Arabic schoolchildren. The Arabic COHIP-SF 19, therefore, can be used to assess subjective oral health needs among Libyan children as part of national surveys and clinical assessment in dental practice. However, the EFA suggested some modifications to the subscales which has been identified as an area of further assessment. Further research is required to investigate the longitudinal validity and responsiveness of Arabic COHIP-SF 19 as well as its performance among children from different age groups.
